# Slip and Fall Incidents at Work: A Visual Analytics Analysis of the Research Domain

**DOI:** 10.3390/ijerph16244972

**Published:** 2019-12-06

**Authors:** Jie Li, Floris Goerlandt, Kai Way Li

**Affiliations:** 1 Department of Safety Science and Engineering, School of Ocean Science and Engineering, Shanghai Maritime University, Pudong New Area, Shanghai 201306, China; lijie_jerry@126.com; 2 State Key Laboratory of Explosion Science and Technology, Beijing Institute of Technology, Beijing 100081, China; 3 Department of Industrial Engineering, Dalhousie University, Halifax, NS B3H 4R2, Canada; floris.goerlandt@dal.ca; 4 Department of Industrial Management, Chung Hua University, Hsin-Chu 30012, Taiwan

**Keywords:** slip and fall, bibliometric analysis, knowledge mapping, citation network, VOSviewer, HistCite

## Abstract

Slip and fall incidents at work remain an important class of injury and fatality causing mechanisms. An extensive body of safety research has accumulated on this topic. This article presents an analysis of this research domain. Two bibliometric visualization tools are applied: VOSviewer and HistCite. Samples of 618 slip and fall related articles are obtained from the Web of Science database. Networks of institutions, authors, terms, and chronological citation relationships are established. Collaboration and research activities of the slip and fall research community show that most contributors are from the United States, with the (now closed) Liberty Mutual Research Institute for Safety the most influential research organization. The results of a term clustering analysis show that the slip and fall research can be grouped into three sub-domains: epidemiology, gait/biomechanics, and tribology. Of these, early research focused mainly on tribology, whereas research on gait/biomechanics and epidemiological studies are relatively more recent. Psychological aspects of slip and fall incident occurrence represent a relatively under-investigated research topic, in which future contributions may provide new insights and safety improvements. Better linking of this research domain with other principles and methods in safety science, such as safety management and resilience, may also present valuable future development paths.

## 1. Introduction

Slips and falls are one of the major sources of injuries and fatalities at work [1], at home [2], and in our leisure activities. For instance, nonfatal slip and fall injuries led to an annual number of lost workdays of approximately 23,800, with an associated cost of about 17.5 million USD [3] in the surface stone, sand, and mining industry in the United States. Examples of other industries where slips and falls pose a significant occupational health and safety hazard include healthcare and social assistance, manufacturing, retail, and transportation and warehousing [4], postal services [5], and merchant shipping [6]. Slips and falls also constitute a significant share of injuries of people in their home environments [7,8], for patients in hospital settings [9] or in a home care environment [10], and during leisure, for instance on board cruise ships [11]. Based on official statistics from different countries [12], falls can be classified as falls to a lower level and falls on the same level [13].

It is not entirely clear when the very first scientific research on slips and falls was performed, but it appears that only a handful of studies was performed before 1970. Carlsöö was one of the pioneers in the study of gait [14]. Perkins demonstrated a slip using a multi-image photo sequence of the foot–floor interaction process [15]. He was also the first one to indicate the peaks when a slip is most likely to occur using a trace of the ground-reaction force ratio. Such a trace was explained in more detail later by Strandberg and Lanshammar [16]. Slip and fall research has been speeding up and blooming from around 1990. Up to now, there is a sizeable body of literature on the topic.

Both incident types are common but present complex topics for scientific inquiry, as they involve many factors traditionally studied in different scientific disciplines. From an interdisciplinary safety science perspective, and for reducing slip and fall related incidents and accidents in practice, it is thus important to obtain a holistic view on the research areas and topics which are involved in the study of these phenomena. Systematic literature reviews are useful for decision makers and safety practitioners to develop evidence-based safety assurance programs, and valuable for scientists to understand the state of the art and work on identified research gaps or uncertainties. Several review articles have been made, addressing specific issues or circumstances. Leclerq [17] reviewed the literature focusing on methods for measuring slip resistance. Kong et al. [18] reviewed risk factors of slip and fall incidents during firefighting operations. Moon and Sosnoff [19] presented a review and meta-analysis of strategies for landing safety during a fall. Hartung and Lalonde [20] presented an overview of work focusing on using non-slip socks for fall prevention among hospitalized older adults. Wilkinson et al. [21] reviewed the literature on strategies used by older adults and their informal carers to prevent falls. Larsson et al. [22] studied the effects of gait speed on the perceived risk of slipping on an icy surface.

While the above-mentioned review articles are insightful and useful to understand specific aspects of slip and fall research, these do not provide insights into the range of topics which are covered in the research domain, and in the structure of this scientific domain in terms of impactful journals, authors, and institutions, and its historic development and recent trends. This is not a shortcoming of these works per se, but a result of the focus, scope, and correspondingly applied methodologies in these review articles. For obtaining insights into the structure of a research domain and to identify high-level trends and gaps, a mapping review methodology is more appropriate [23]. Bibliometric analysis and related visual analytics software present appropriate methodologies for such purposes. Bibliometrics is a scientific discipline employing a quantitative analysis methodology to explore the nature of scientific activities. It was coined by Pritchard [24] and was later elaborated by Cole and Price [25,26]. Through visualization of processed bibliometric data, the research domain can be explored in a visual analytics approach, from which insights in the structure of the research domain can be obtained.

Acknowledging the value of such insights in understanding the domain of interest as a whole, and for advancing it, several safety-related bibliometric analyses have been reported in the literature. For instance, Valero and Monk [27] presented a bibliometric analysis of human–computer interaction (HCI). Other bibliometric analyses focusing on human factors and ergonomics can also be found in the literature [28,29]. Several visualization-focused bibliometric analyses have also been presented in the safety science literature. Examples include the work by van Nunen et al. [30] on safety culture research, Jin et al. [31] focusing on the construction safety literature, and Yang et al. [32] addressing university laboratory safety. The method has also been used to gain insights into the structure of the *Safety Science* journal [33] and in analysing knowledge communication between core safety science journals [29].

Even though slips and falls have been studied since the 1970s, leading to an extensive body of literature, a bibliometric study focusing on slips and falls at work has not been reported. This paper aims to fill this gap, to enable high-level insights in and understanding of the developments and knowledge distribution of this scientific research domain. Similarly, e.g., in [30] and [33], specific focus is given to the publication trends in this research domain, impactful authors and institutions, and terms analysis. A chronological analysis is performed as well, providing insight in the historical development of the research domain. This knowledge is primarily useful for researchers working in this area (especially early career researchers relatively new to the research domain), to gain understanding of the structure of the research domain, to identify trends and emerging topics, and possible research collaborators. For funding agencies, knowledge about current topics and gaps identified in the research domain, can be helpful to support decisions on what research proposals to fund. In addition, the author network can assist funding agencies in appreciating the importance of specific authors within the research community.

The remainder of this article is organized as follows. Section 2 describes the data, and includes high-level statistical analyses and metrics providing preliminary insights in the research domain. The section also introduces the methods used in the bibliometric analysis and the software used for visual analytics. Section 3 presents the results, including an author analysis, institution analysis, terms analysis, and a chronological citation network analysis. A discussion is provided in Section 4, and conclusions are made in Section 5.

## 2. Data and Methods

### 2.1. Data Collection and Preliminary Analysis

**Figure 1:**
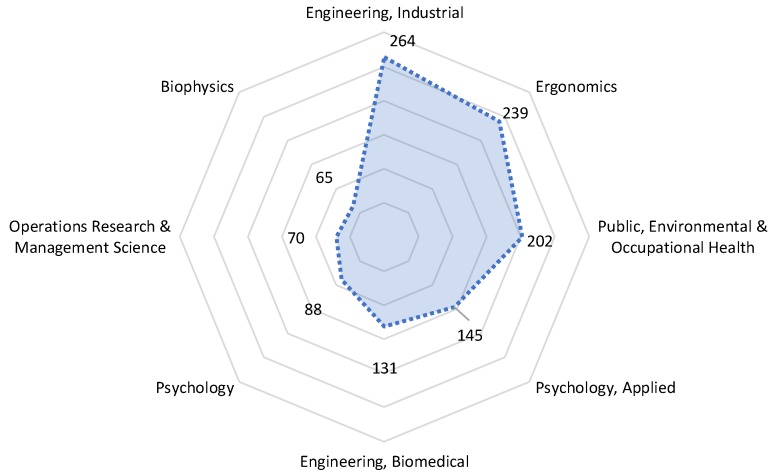
The distribution of the sample data based on the Web of Science Categories.

The scientific literature on slip and fall incidents and accidents was downloaded from the online version of Web of Science Core Collection, which is one of the leading citation databases and includes high-quality bibliographic information about publications in a wide range of scientific disciplines. Consequently, it has been widely used in bibliometric review research [34,35]. In searching the database for slip and fall research, the following keywords were entered in the Web of Science search portal: “floor slipperiness” OR “floor slip resistance” OR “floor coefficient of friction”; OR “floor COF”; OR “slip*” and “fall*”. The timespan was set to all years, i.e., the entire timespan of the database is included. A total of 2366 publications were obtained based on the data selection criteria. Then the results were refined by using the Web of Science Categories of the publications, where articles in irrelevant categories such as Geosciences, Multidisciplinary and Geochemistry and Geophysics were removed from the results list. Figure 1 shows that the distribution of the sample data originates primarily from following categories: Engineering, Industrial (264), Ergonomics (239), Public, Environmental & Occupational Health (202), Psychology, Applied (145), Engineering, Biomedical (131), Psychology (88), Operations Research & Management Science (70) and Biophysics (65). Finally, 618 target publications were downloaded and analysed in the present research. It should be noted that, in the Web of Science Categories, one paper may belong to two or more categories. Thus, the sum of the number of papers in each category can be larger than 618, the total number of publications in the dataset.

**Figure 2:**
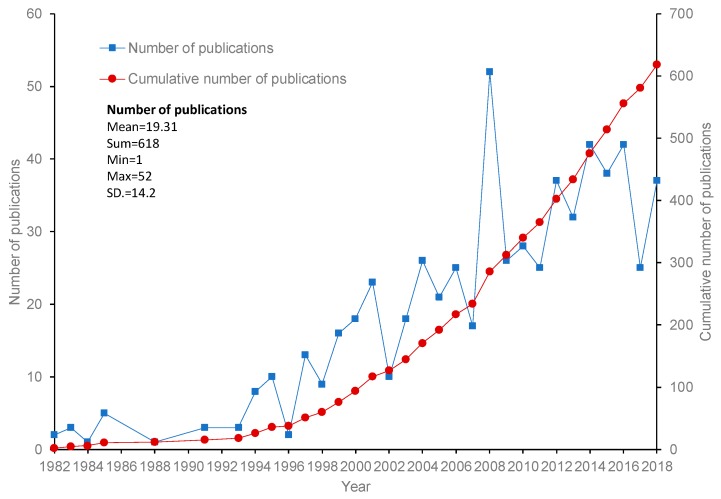
Annual publications in the slip and fall research domain, based on the sample data.

The annual trend of research outputs is a simple but insightful way the to show the global activity and scientific attention to slips and falls. The time series of the published slip and fall papers is shown in Figure 2. It shows that the international slip and fall incidents research community does not publish very large numbers of papers each year, which corresponds to the relatively small number of research groups addressing this topic. There is a clearly increasing trend in the number of articles published in the research domain between 1982 and 2018, even if the data for 2018 includes only articles published before October 2018. Based on the high-level annual publication trend, three research eras can be distinguished in the slip and fall research domain. Before 1998, the number of articles published annually was below 15. Between 1998 and 2008, the number of contributions saw a steady and relatively rapid increase, reaching a peak in 2008 with 52 papers. After 2008, there was a decrease in the number of outputs, but the annual number of contributions almost always has a higher value than in the preceding eras.

### 2.2. Bibliometric Methods and Visualization Tools Used in the Analysis

Bibliometric mapping methods and tools were used in this study, where scientific data was visualized using various processes. Bibliometric mapping as a research method is the application of quantitative methods for visually representing scientific literature based on bibliographic data.

The VOSviewer software was applied in the research to visually represent the bibliometric map of the slip and fall research domain. As software for analysing bibliometric networks with text mining functionalities and advanced visualization options, it has been widely used for analysing various knowledge domains (Publications on applications of VOS viewer, https://www.vosviewer.com/publications), including topics addressing environment and public health [36,37]. This software was developed by Nees Jan van Eck and Ludo Waltman from Leiden University in the Netherlands. It is a free and widely used bibliometric mapping tool for constructing and visualizing bibliometric networks including journals, authors, and individual publications [38]. The networks can be constructed based on co-citation, bibliographic coupling, or co-authorship relations [39,40,41]. VOSviewer also offers text mining functionalities which can be used to construct and visualize co-occurrence networks of important terms extracted from the investigated body of scientific literature [42]. Usually, terms are extracted from the titles and abstracts of the articles in the dataset. In the present paper, VOSviewer was used to analyse the authorship and terms clusters of the international slip and fall research domain.

In addition, the HistCite software tool was also applied to analyse the citation network for slip and fall studies. HistCite is a software implementation of *algorithmic historiography*, and is developed by Garfield(HistCite Help, https://edisciplinas.usp.br/pluginfile.php/4428395/mod_page/content/118/Manual_histCIte_bom.pdf), who is also the father of the Web of Science database [43]. It allows presentation of a citation network of highly cited articles during a certain period, thus mapping out the evolution path of the scientific domain in focus.

**Figure 3:**
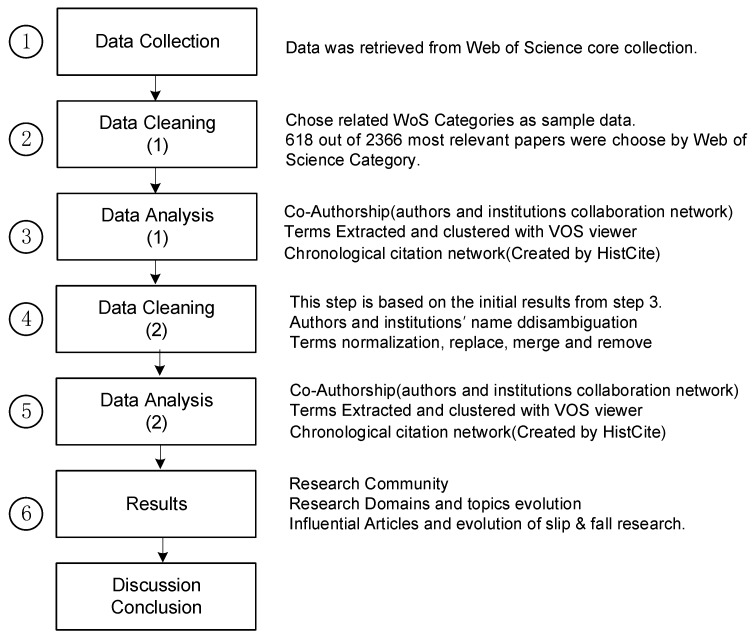
Flowchart illustrating the process sequence applied in this research.

In order to conduct and create the bibliometric analyses and visualizations of the slip and fall research domain, the steps presented in Figure 3 were followed. First, as explained in Section 2.1, the slip and fall papers from the Web of Science were identified and extracted. The citation data of this dataset contained authors, institutions, titles/keywords/abstracts, and references, for each record. Second, irrelevant records were removed based on the categories of the papers, see also Section 2.1. Third, an initial analysis of the data was performed, to identify possible errors or ambiguities in the data (e.g., author name disambiguation), so that a data cleaning process could be completed. The fourth and fifth steps were similar to steps 2 and 3, the second-round data cleaning in this step was based on the initial analysis in step 3. The sixth step concerned the construction of figures to visualize the author and institution networks, the term maps, and the history of citation network figure. Finally, the results were discussed, and conclusions were drawn.

## 3. Results 

### 3.1. Authors Analysis

**Figure 4:**
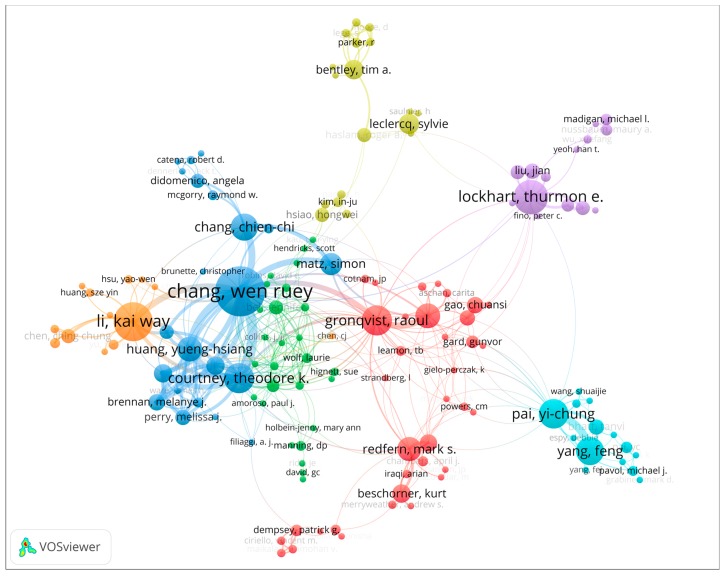
Clusters in the co-authorship network of slip and fall research.

**Figure 5:**
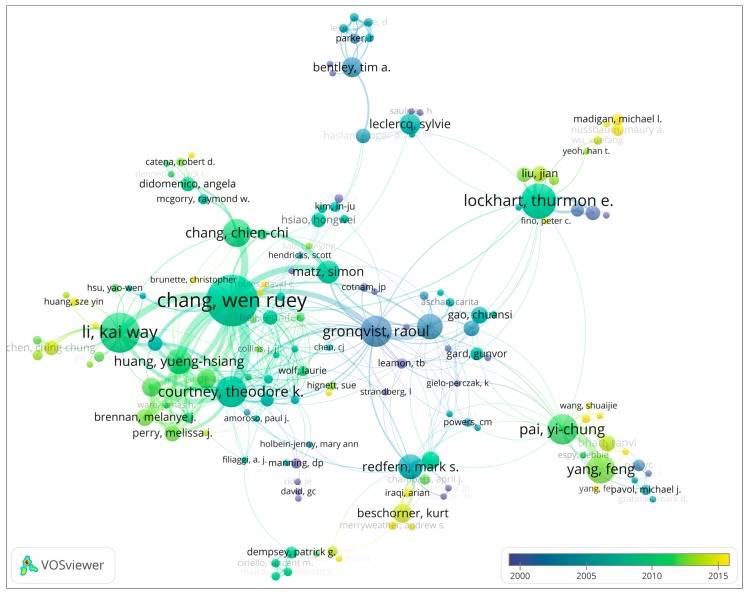
Authors’ average publication years in the co-authorship network of slip and fall research.

Authors active in the slip and fall research domain are knowledge providers, where especially the highly productive authors have a possibly influential role. An author’s collaboration analysis not only can display the core leading knowledge providers, but also the social networks among these authors. This is interesting knowledge, for instance, for early career researchers entering this research domain, or for external stakeholders seeking advice from world-class experts. In order to obtain a visually clear authors’ collaboration network, not all authors are included in the network. In the current research, authors who have published more than two articles on slip and fall incidents or accidents are included in the collaboration network in Figure 4. The average year of publication was also determined and displayed in the network shown in Figure 5. In both figures, the size of the nodes indicates the number of publications for each author. The lines connecting the nodes indicate collaboration between the authors. The color of a node in Figure 4 signifies the clusters in which this node is located, where clusters represent networks of authors whose work is linked through co-authorship relations. The colors in Figure 5 show the average publication year of each author. Detailed information of the highly productive authors, their average publication years, and total citations are also listed in Table 1.

The productivity of authors in a given research domain is one of the most important indicators to measure their impact in that area. The results of the author outputs show that W.R. Chang of the Liberty Mutual Research Institute for Safety (LMRIS), published 58 papers and ranked first place in this area. He is followed by Li (38), Lockhart (31), Courtney (25), Gronqvist (25), Pai (24), Yang (22), Chang, C. C. (21), Hirvonen (18), and Huang (18). As listed in Table 1, LMRIS has more productive authors than any other institution, signifying that this institution and its researchers play a very important role in the international slip and fall research community.

Authors can be divided into different groups based on the strength of their collaboration. Each group of authors shows the different sub-communities of the slip and fall research domain. For example, W. R. Chang has the largest node in his group, and has 42 collaborators in the global collaboration network. Li, Lockhart, Gronqvist, and Hirvonen also play leading roles in their group. Figure 5 shows the average publication year of each author. On the individual level, the most recent active authors in slip and fall research are Nussbaum (2017), Madigan (2016), Beschorner (2013), Chen (2013), Bhatt (2013), and Liu (2013). These authors are not only highly productive but have been active particularly in recent periods.

### 3.2. Institutions Analysis

**Figure 6:**
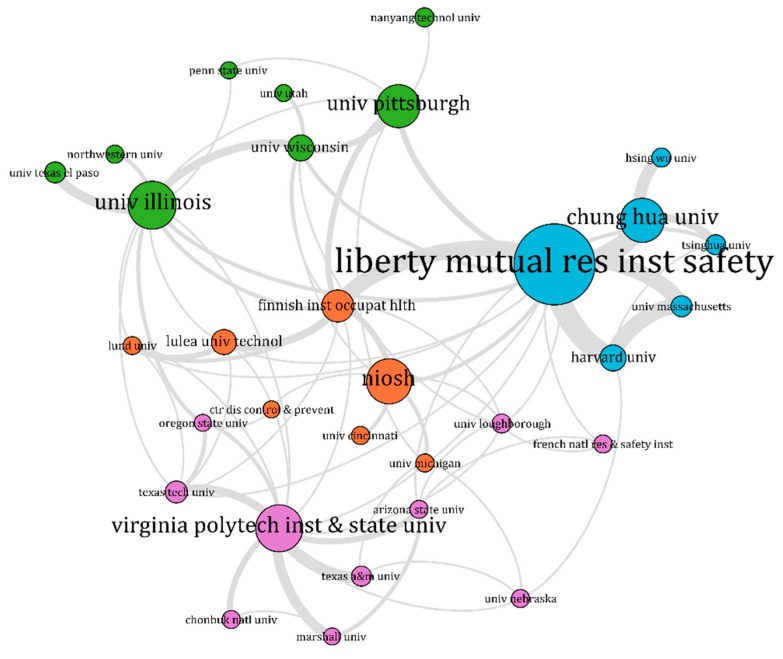
Clusters in the institutions’ collaboration network in slip and fall research; the figure was analysed by VOSviewer, and visualized by Gephi [44].

**Figure 7:**
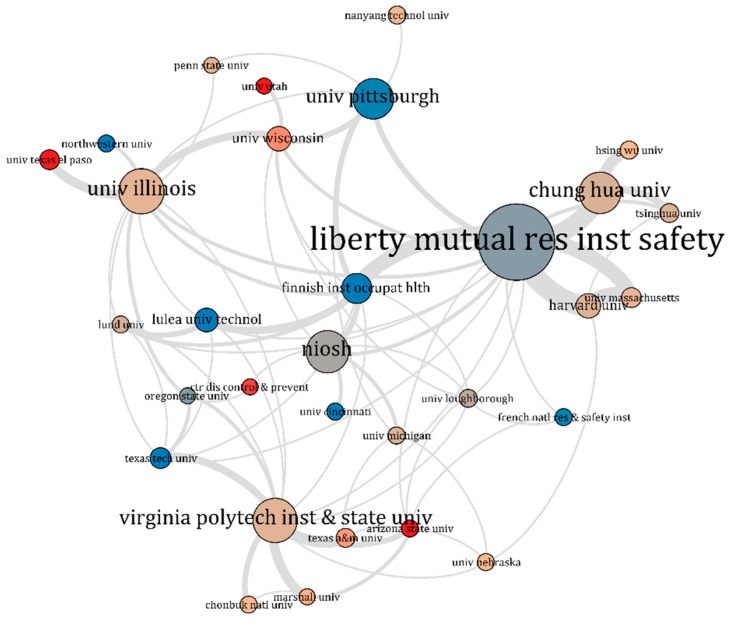
Institutions’ average publication years in the collaboration network of slip and fall research, red indicates more recent contributions, blue indicates older contributions (the figure was analysed by VOSviewer and visualized by Gephi).

Figure 6 and Figure 7 show the cooperation networks of the institutions which published more than five articles on slip and fall incidents and accidents, including the average publication years of these institutions. Similarly, as in Section 3.1, Figure 6 shows the clusters of the institutions based on the strength of their connections, whereas Figure 7 shows the active research institutions in recent years. The size of the nodes in these network shows the number of articles the institutions have published in the journals included in the data sample. The lines connecting the nodes indicate collaboration among the institutions. Nodes with the same colour indicate that the institutions have more collaboration in slip and fall research than others. Detailed information of the institutions included in the network is listed in Table 2.

Among these institutions, the LMRIS was the most productive, having published 69 papers. It was followed by the University of Illinois (36), Virginia Polytechnic Institute and State University (35), National Institute for Occupational Safety and Health of the United States (NIOSH, 33), Chung Hua University (32), and University of Pittsburgh (31). As is known from the analysis of the slip and fall authors in Section 3.1, LMRIS had a group in slip and fall research, which played a primary role in the research domain. There were 16 links between LMRIS and other institutions, showing its strong collaboration with many other institutions.

### 3.3. Terms Analysis

The VOSviewer software was used to apply the so-called Automatic term identification method [42] to extract the terms, noun phrases, or terminologies related to the research topic from bibliographic data. In the present work, slip and fall related terms were extracted from the title, abstract, and keywords of the publications obtained in the dataset in Section 2.1. Setting a restriction to include terms which appeared at least five times, a total of 457 terms was extracted, mapped, and clustered in two-dimensional space.

**Figure 8:**
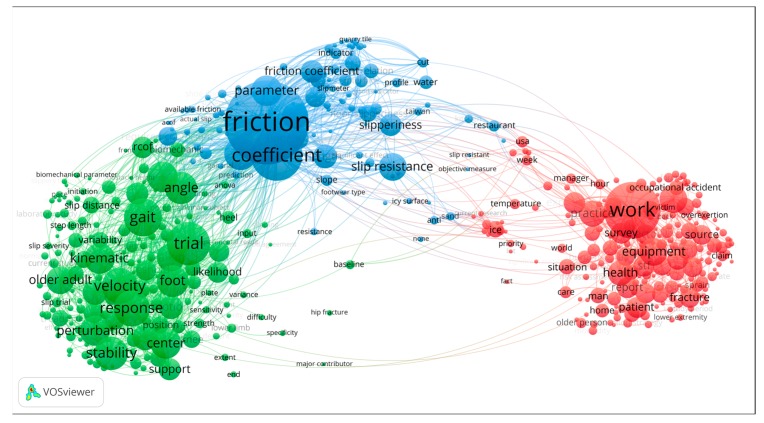
Terms cluster of the slip and fall research domain.

The cluster of the terms for the slip and fall research domain is shown in Figure 8. These terms can be classified into three clusters, based on the connective strength of these terms. Cluster #1, displayed by red dots, includes terms commonly found in epidemiology and incident occurrence analysis of slips and falls. Cluster #2, shown by green dots, includes those terms commonly found in studies in gait or biomechanical studies, which typically focus on finding causes of slip and fall incidents and accidents. Cluster #3, indicated by blue dots, includes terms commonly found in tribology studies, and typically concern friction measurement and determination of the friction coefficient at the foot–floor interface. The major terms in each cluster are listed in Table 3. 

**Figure 9:**
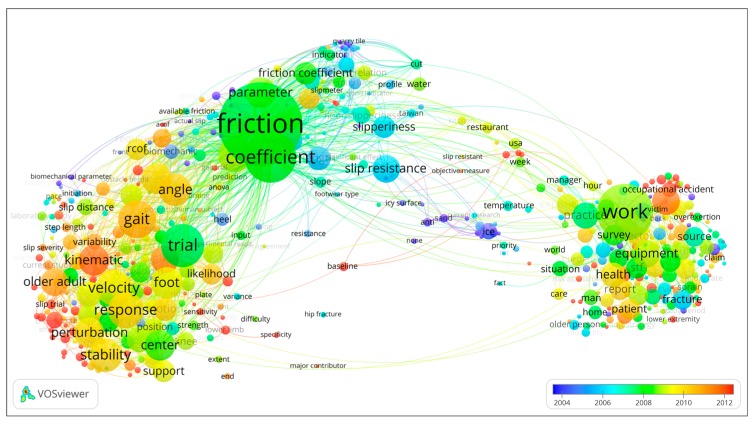
Terms average year distribution of slip and fall research domain.

Figure 9 shows the same network as in Figure 8, except that it shows a time factor of the terms. The average publication year when a term appeared in the research domain was calculated and then added to each node in the map. The warmer (redder) the terms, the more recently the terms appeared. Comparing the time factor in the three clusters, it shows that terms appearing in cluster no. 1, epidemiology and slip and fall incidence, and no. 2, gait or biomechanical, are more recent. Understanding the reasons for the occurrence of slip and fall incidents represents an important part of the slip and fall research domain. Friction measurement and determination of the friction coefficient constitute an important element in the causation of slips and falls. Hence, it has a long history of activity in this research domain, paying attention to environmental factors such as ice, water, slope, and footwear type. In recent years, more research has been conducted in the gait or biomechanical cluster, which is part of addressing the human factors in the occurrence of slip and fall incident and accident occurrence.

### 3.4. Chronological Citation Network

The number of times a paper has been cited by other papers, either within the research domain under scrutiny, or in the entire scientific body of literature, has been widely adopted in bibliometric analyses to quantify the impact and influence of the paper [30,31].

In this section, not only are the highly cited articles in the slip and fall research domain identified. Rather, also the networks between these highly cited papers are determined, by using the HistCite software. HistCite distinguishes two types of citation scores: the local citation scores (LCS) and global citation scores (GCS). The local citation score of an article is the number of citations of this article within the local database being studied (which here means the database on slip and fall research, containing 618 articles as introduced in Section 2.1). The global citation score is the total number of citations, including those outside the studied database [45,46]. For example, the citation score counted in the Web of Science database is a global citation score. This score reflects the overall impact of an article in a certain scientific database, whereas the local citation score shows the impact of an article in the specific domain. In the study of a specific research domain, local citation scores are more appropriate than global citation scores, as they provide information about the importance of a research contribution within its research domain context. Hence, in this paper, the local citation score was used as one of the parameters to select papers and construct the citation network. For both the global citation score and the local citation score, based on the functionalities of HistCite [43], no distinction was made between self-citations (i.e., citations to an article by the same author) and citations from other authors.

**Figure 10:**
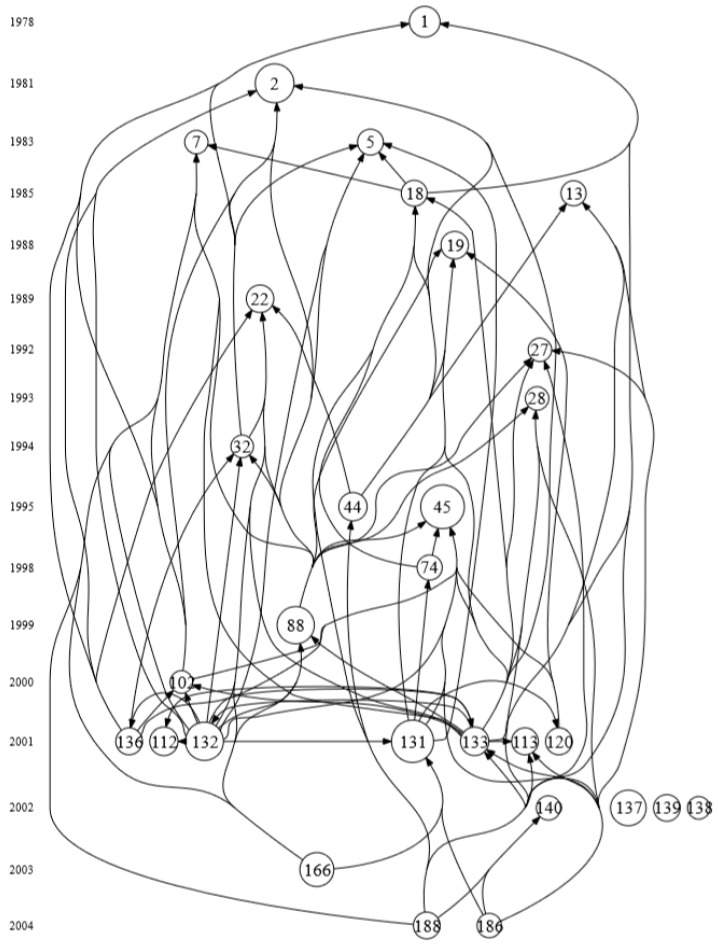
Chronological citation network among highly cited slip and fall publications.

To obtain a more complete chronological network of the slip and fall research domain, papers which have been cited more than 20 times in the references of the citing articles were also included and used for constructing a citation network. This means that 27 papers were added to this network. Finally, the top 30 highly cited slip and fall papers were extracted and used to construct a citation network. Figure 10 shows the chronological citation network of the slip and fall research domain. There are 30 nodes and 88 links in this network. The number of citations of the papers in the network ranges from 30 to 106. The nodes indicate a paper in the network, where the numbers associated with these nodes’ links to detailed information about the corresponding articles, elaborated in Table 4. These highly cited papers originated from nine different journals. Sixteen of these were published in *Ergonomics*, five were published in *Safety Science* (*J Occup Accid* is the former name of *Safety Science*), with two published in *Applied Ergonomics* and the *Journal of Biomechanics*. In relative terms, 53.3% of the highly cited articles are published in *Ergonomics*, reflecting its status as the leading journal in the slip and fall research domain, and indicating that slips and falls are considered an important subdomain in ergonomics. The time distribution of the papers shows that seven (23.3%) highly cited papers were published in 2001, and 71.4% (5/7 = 71.4%) of these papers from 2001 were published in *Ergonomics*. Chang W. R. (4), Cham (3), Rosqvist (3), Strandberg (3), and Redfern (2) are influential authors who published more than one highly cited article in the slip and fall citation network.

The citation network also shows the evolution of slip and fall research from 1978 to 2004. Within this network, Perkins was the first highly cited article with 53 citations [15], with a research focus on the measurement of slips between the shoe and the ground during walking. This article can be regarded as one of the most important and influential papers in the history of the slip and fall research domain. Even though Carlsöö can be regarded as a pioneer in this research area as indicated in Section 1 [14], his paper only received 4 citations from the local dataset and is therefore not included in the network. Among the articles, Leamon and Murphy [47] studied the relationships between incidence and cost of falls and age, gender, industry, and other factors, and discussed the costs and financial burdens of slip and fall incidents in the United States. The work received 106 local citation scores, making it the most cited article in the network. Among the citation network, papers 137 [48], 138 [49] and 139 [50] (Figure 10) are three special papers. They do not connect with any other articles in the current network. Paper 137 focuses on quantifying changes in gait biomechanics when subjects anticipate slippery environments. Paper 139 reports the heel contact dynamics during slip events, while paper 138 concerns strategies for dynamic stability during locomotion on a slippery surface. The whole chronological citation network shows the main evolution of slip and fall research, including fall incidents and accident occurrence, slip resistance, friction and gait biomechanics, and psychological research related to perceptions in slip and fall contexts.

## 4. Discussion

In the collaboration network analysis, both authors and institutions publishing work in the slip and fall research domain were included. It should be noted that authors are associated with institutions. Therefore, there were significant correlations between the networks of authors and institutions. For example, the LMRIS was the most productive institution within the research domain. Most of the highly influential authors in Table 2 (7/17) were fully or partially affiliated with the LMRIS. Therefore, it can be considered most unfortunate that LMRIS was shut down in June 2017 [75], and its scientific research in the slip and fall research area was terminated. The increasing trend of the annual number of publications seems to have paused in 2017, which might also be attributed, at least partially, to the shutdown of LMRIS. The outputs and collaborations in slip and fall articles shown in Figure 4 to Figure 7 show that the research community is comprised of a relatively small group. This group has been comprised of professional scientists and scholars from a limited amount of institutions.

The dominance of research performed in the United States is remarkable, and it may be questioned if the same clusters or trending topics would be found if only authors or institutions from the United States, or from other countries were considered. Focusing on the United States, given the dominance of the highly influential authors from that area, it is likely that the results would not change significantly if only work originating from the United States were included. Perhaps the centrality of some authors or institutions would shift somewhat, and the relative occurrence frequency of topics could change somewhat. For other countries, with fewer contributions, it may well be that significantly different patterns would be found. For instance, it may be hypothesized that authors from Finland or Sweden would be comparatively more focused on friction measurement in icy conditions, or the influence of snow or ice on biomechanics in the context of slips and falls. Such more detailed questions were however not further analysed in this paper, and were left for future research. A note is in place on the data collection method, and related limitations of the presented work. The slip and fall focus in this paper was the slip and fall of humans, which implied a subsequent contact with the ground, with likelihood of injury or fatality of the victim. In searching the articles in the literature, and in constructing the dataset using the keywords as described in Section 2.1, the keywords related to “slip and fall” could also occur in other scientific disciplines such as those focusing on the slipping and falling of land and objects in earth science or physics. Initially, a total of 2366 publications were initially obtained based on the chosen selection criteria. However, only 618 articles were retained as many articles were found irrelevant based on the Web of Science Categories. It is possible that some slip and fall articles could still appear in categories which do not correspond to the focus on human slip and fall incidents. It is also possible that there are some omissions in the constructed dataset, in the sense that other research work has in fact been performed, but not included in the dataset. This related to a commonly known challenge in bibliometric studies of particular research domains, where it is difficult to keep a balance between including several keywords and minimizing the number of articles included in the initial dataset which needs manual screening to obtain the finally analysed dataset. This was a limitation of the study. 

Another point concerns the clustering of the research as presented in Figure 8. The clustering algorithm implemented in the VOSviewer software is based on the method of community detection and modularity optimization [76]. This approach allows partitioning the elements in the network into *n* clusters, where *n* is greater than or equal to two. Here three clusters of slip and fall topics were identified. It appears that the slip and fall research community contains more physical ergonomists then psychologists. Hence, more focus by psychologists may be required to strengthen the scientific understanding of perceptual and psychophysical aspects of slip and fall incidences. It may also be worthwhile to explore and strengthen links between the slip and fall research community and other safety principles and approaches, for instance, safety management, risk analysis and uncertainty based decision analysis, and resilience engineering.

It should also be noted that all scientometric analyses use citations from all authors, i.e., also self-citations are counted. It is possible that in the local and global citation analyses of Table 4, some authors disproportionally cited their own work, which may also skew the results (e.g., from the chronological citation network of Figure 10). The full counting of all citations is an assumption embedded in the applied HistCite software, see [43]. It is left for future research for the scientometric mapping research community to develop methods which distinguish self-citations from citations from other authors. Consequently, the stability of the findings presented here can be further elucidated.

The chronological citation network analysis shows the citation connections between highly cited research from 1978 to 2004. More than 50% of these articles were published in the *Ergonomics* journal. The year 2001 is special, in the sense that it produced the most highly cited papers (Figure 10), while simultaneously displaying one of the peaks in the annual publication curve. With the development and evolution of slip and fall research, a shift of the centre point of research attention can be identified, from friction and tribology early on, to gait and biomechanics later on, and more recently to psychological aspects.

The quantification-based scientometric analysis methods applied in this work clearly lead to various insights into the research domain. However, these methods are limited in the sense that they only provide high-level insights in the domain. Qualitative insights from the research studies, such as limitations of particular friction measurement methods, or what the benefits and downsides are of certain biomechanical behaviour in particular contexts, cannot be obtained using these methods. Qualitative reviews would also be needed to provide deeper insights, e.g., which highly influential authors of Figure 4 are active in what research topic clusters, and if emerging authors from Figure 5 also work on new topics as found in Figure 9. Currently, scientometric mapping methods cannot provide such insight. Hence, other review methods, such as critical reviews, meta-analyses, or systematic review methods, are more appropriate for obtaining more qualitative insights, see [23] for an overview of review methods and their strengths and limitations. However, such qualitative reviews are outside the scope of the current work, and are left for future research.

Apart from providing insights into the results per se, and on the limitations of the study, it is also instrumental to consider how the results may be used. Consider for example an early career researcher who aims to perform research on slips and falls, for instance, a first-year graduate student. He or she needs to get familiar with the research domain. From Figure 4, he or she can find the main authors in the research domain, and in Figure 5 the more recently active researchers. This can be useful to know which authors to follow, read articles from, or suggest as reviewers. Table 3 and Figure 9 can give inspiration as to what are trending topics in the research domain, and can also indicate areas where not much research has been done, and, hence, where new research directions can be explored. As indicated above, perceptual and psychological issues may be such new directions, as can the link between slip and fall and other safety principles. Figure 10 and Table 4 can help the student to identify those articles with which an expert in the domain should likely be familiar, and the chronological mapping can help in deciding in which order to read what works. Another example of how the results can be used can be an experienced researcher (e.g., tenured faculty) who wants to establish a collaboration with an active researcher in the domain, or perhaps hire an emerging scholar in the domain. Here, the results of Figure 5 can be instrumental. As a final example, a funding agency may want to decide on the leading topics for a new research program addressing slips and falls, exploring new areas or strengthening emerging domains. As already indicated above, Table 3 and Figure 9 can be starting points for identifying trending topics and areas where no or only very little research has been performed.

## 5. Conclusions

In this article, a visual analytics analysis was presented of the research domain focusing on slip and falls at work. Using the Web of Science database as a data source, publication trends were identified, where it was found that the research domain started in the 1970s and gained increasing attention especially since the late 1990s. Impactful authors and institutions were identified, along with their collaborations. Here, it was found that several authors from the (now defunct) Liberty Mutual Research Institute for Safety were amongst the most influential in the field, with Wen Ruey Chang the most impactful author overall. By far the most work originated from the United States, with Taiwan, Finland, and Sweden other significantly contributing countries. A terms analysis revealed three major clusters: epidemiology and slip and fall incidence, gait of biomechanics, and friction measurement and coefficient. An analysis of the average years in which these terms were found in the literature revealed that research recently has focused more on the gait and biomechanics cluster, indicating that more work is recently being performed on addressing the human factors in the slip and fall occurrence. The results also suggest that more work on the perceptual and psychological aspects of slip and fall occurrence would be beneficial. Another area of future research, which has not attracted significant attention, is the link between slips and falls, and other (more generic) safety principles such as safety management, risk analysis and uncertainty based decision analysis, and resilience engineering. A local and global citation analysis revealed which articles have been most influential within the slip and fall research community, and a chronological citation network showed how the field has evolved in light of the interconnection between these highly influential articles. Finally, a discussion has indicated how these results can be used by researchers and funding agencies, addressed some limitations of the current work, and made suggestions for future research.

## Figures and Tables

**Table 1:** Bibliometric network and citation information of authors who published more than 10 articles in the slip and fall research domain.

Rank	Authors	Institutions	Links	TLS	NP	TC	APY
1	Chang, W.R.	LMRIS	42	195	58	730	2008.62
2	Li	Chung Hua Univ.	21	76	38	403	2009.95
3	Lockhart	Virginia Tech	19	47	31	688	2008.77
4	Courtney	LMRIS	31	137	25	578	2007.96
5	Gronqvist	Finnish Inst. Occupat. Hlth.	34	86	25	695	2002.16
6	Pai	Univ. Illinois	20	49	24	602	2010.75
7	Yang	Univ. Illinois	8	30	22	328	2012.05
8	Chang, C.-C.	LMRIS	9	42	21	190	2010.05
9	Hirvonen	Finnish Inst. Occupat. Hlth.	15	42	18	436	2002.83
10	Huang	LMRIS	14	92	18	199	2010.50
11	Redfern	Univ. Pittsburgh	18	36	17	783	2005.12
12	Matz	LMRIS	8	35	15	186	2008.33
13	Leclercq	French Natl. Res. & Safety Inst.	6	8	13	151	2006.15
14	Bentley	Massey Univ.	8	23	12	308	2003.00
15	Beschorner	Univ. Pittsburgh	7	17	11	66	2013.73
16	Lombardi	LMRIS	17	76	11	139	2011.27
17	Verma	LMRIS	10	66	11	105	2011.27

Links means the number of institutions that link the selected node with others. The stronger the links, the more patterns can be identified in the network. Total link strength means the weighed links of the selected nodes. Citations indicates the number of times an author is cited within the article sample of the slip and fall research domain. TLS, total links strength; NP, number of papers; TC, total citations; APY, average publications year. LMRIS, Liberty Mutual Research Institute for Safety.

**Table 2:** Bibliometric network and citation information of the top 10 most productive institutions in the slip and fall research domain.

Rank	Institutions	Countries/Regions	Links	TLS	NP	TC	APY
1	Liberty Mutual Res. Inst. Safety	USA	16	55	69	1397	2007.80
2	Univ. Illinois	USA	12	23	36	750	2011.36
3	Virginia Polytech Inst. & State Univ.	USA	13	30	35	671	2011.23
4	NIOSH	USA	7	12	33	709	2008.76
5	Chung Hua Univ.	TAIWAN	3	20	32	340	2010.69
6	Univ. Pittsburgh	USA	7	13	31	853	2005.94
7	Finnish Inst. Occupat. Hlth.	FINLAND	10	26	20	609	2004.25
8	Harvard Univ.	USA	4	21	14	181	2010.93
9	Univ. Wisconsin	USA	7	14	14	108	2014.57
10	Lulea Univ. Technol.	Sweden	6	11	13	253	2005.15

TLS, total links strength; NP, number of papers; TC, total citations; APY, average publications year. NIOSH, National Institute for Occupational Safety and Health (US)

**Table 3:** Clusters of terms in slip and fall articles.

Title of Each Cluster	Selected Terms in Each Cluster (Occurrences of a Term)	Legend	Size of The Cluster
1# epidemiology and slip and fall incidence	Work (89), equipment (41), occupational injury (41), practice (38), health (36), experience (31), patient (30), report (30), STF(Slips, trips, and falls, 30), survey (30), effort (29), database (28), fracture (28), nature (28), review (28), sector (28), source (28), day (26), fatality (26).	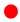	192
2# gait or biomechanical	Trial (66), gait (57), response (55), velocity (54), stability (50), angle (48), foot (47), center (45), kinematic (45), speed (45), perturbation (44), older adult (41), walking (41), mass (37), recovery (36), motion (34), ground reaction force (32), support (32), RCOF (required coefficient of friction, 31).	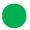	165
3# friction measurement and coefficient	Friction (138), coefficient (81), experiment (49), parameter (45), slip resistance (41), COF (coefficient of friction, 38), length (34), friction coefficient (33), slipperiness (32), floor surface (30), surface condition (29), required coefficient (26), floor slipperiness (24), contaminant (22), friction measurement (21), correlation (20), water (19), interface (17), regression model (17).	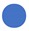	100

**Table 4:** Timeline for the history of slip and fall research.

RY	#	First Author	Year	Source	LCS	RLCS	References
1	1	Perkins	1978	*Astm Stp*	53	8	[15]
2	2	Strandberg	1981	*J Occup Accid*	83	3	[16]
3	5	Strandberg	1983	*Ergonomics*	38	19	[51]
4	7	Perkins	1983	*Ergonomics*	31	27	[52]
5	13	Strandberg	1985	*Ergonomics*	36	21	[53]
6	18	Tisserand	1985	*Ergonomics*	37	20	[54]
7	19	Manning	1988	*J Occup Accid*	43	12	[55]
8	22	Gronqvist	1989	*Ergonomics*	43	13	[56]
9	27	Swensen	1992	*Hum Factors*	32	25	[57]
10	28	Myung	1993	*Int J Ind Ergonom*	32	26	[58]
11	32	Redfern	1994	*Ergonomics*	30	28	[59]
12	44	Gronqvist	1995	*Ergonomics*	45	9	[60]
13	45	Leamon	1995	*Ergonomics*	106	1	[47]
14	74	Bentley	1998	*Ergonomics*	35	22	[61]
15	88	Hanson	1999	*Ergonomics*	79	5	[62]
16	103	Brady	2000	*J Biomech*	30	29	[63]
17	112	Chang W.R.	2001	*Ergonomics*	44	10	[64]
18	113	Chang W.R.	2001	*Ergonomics*	39	15	[65]
19	120	Kemmlert	2001	*Appl Ergon*	39	16	[66]
20	131	Courtney	2001	*Ergonomics*	98	2	[67]
21	132	Redfern	2001	*Ergonomics*	81	4	[68]
22	133	Gronqvist	2001	*Ergonomics*	44	11	[69]
23	136	Cham	2001	*J Biomech*	39	17	[70]
24	137	Cham	2002	*Gait Posture*	70	6	[48]
25	138	Marigold	2002	*J Neurophysiol*	30	30	[49]
26	139	Cham	2002	*Safety Sci*	40	14	[50]
27	140	Chang W.R.	2002	*Safety Sci*	34	23	[71]
28	166	Lockhart	2003	*Ergonomics*	64	7	[72]
29	186	Chang W.R.	2004	*Appl Ergon*	33	24	[73]
30	188	Li	2004	*Safety Sci*	39	18	[74]

Note: RY is short for ranked by publication year of the paper, # is the unique number of the papers in the network, LCS is for local citation score, RLCS is ranked by LCS. *J Occup Accid* is the former journal name of *Safety Science* before 1990.
